# Multivariate pattern analysis of medical imaging-based Alzheimer's disease

**DOI:** 10.3389/fmed.2024.1412592

**Published:** 2024-07-19

**Authors:** Maitha Alarjani, Badar Almarri

**Affiliations:** Department of Computer Science, College of Computer Sciences and Information Technology, King Faisal University, Al-Hofuf, Saudi Arabia

**Keywords:** Alzheimer's disease, blood flow, multivariate pattern analysis, fMRI, neuroimaging, biomarker

## Abstract

Alzheimer's disease (AD) is a devastating brain disorder that steadily worsens over time. It is marked by a relentless decline in memory and cognitive abilities. As the disease progresses, it leads to a significant loss of mental function. Early detection of AD is essential to starting treatments that can mitigate the progression of this disease and enhance patients' quality of life. This study aims to observe AD's brain functional connectivity pattern to extract essential patterns through multivariate pattern analysis (MVPA) and analyze activity patterns across multiple brain voxels. The optimized feature extraction techniques are used to obtain the important features for performing the training on the models using several hybrid machine learning classifiers for performing binary classification and multi-class classification. The proposed approach using hybrid machine learning classification has been applied to two public datasets named the Open Access Series of Imaging Studies (OASIS) and the AD Neuroimaging Initiative (ADNI). The results are evaluated using performance metrics, and comparisons have been made to differentiate between different stages of AD using visualization tools.

## 1 Introduction

The human brain is a highly complex organ regulating the human neurological system. The human neocortex has up to 100 billion neurons connecting throughout the brain (1). They constitute a vast, interconnected network linked to human activities and emotions. Various neuroimaging techniques can acquire a wide range of brain signals. The term "neuroimage" is based on the representation of brain functionality or architecture (2). AD is among the most common types of memory loss in the twenty-first century and is a significant healthcare problem. As per statistics, there are ~5.5 Americans aged 65 years and older affected by AD (3). AD is a progressive brain disease. It is marked by a loss of executive function that treatment cannot resolve. Thus, studies have been conducted to develop ways to predict the disease, especially before symptoms appear, to slow or prevent them from worsening (4).

Traditionally, AD was detected through an invasive technique. Recently, multiple neuroimaging modalities have been developed to identify AD: positron emission tomography (PET) uses specific radiotracers to visualize and quantify amyloid plaques in the brain; electroencephalography (EEG) is utilized to obtain the electrical activity; and functional magnetic resonance imaging (fMRI) is utilized to measure the functionality of the brain with the help of oxygen level change detection in various parts of the brain, such as voxels (5). Moreover, the anatomical brain features are studied using magnetic resonance imaging (MRI), having high spatial determination, and can compare soft tissues (6).

Because neuroimaging techniques are rapidly changing, combining large amounts of high-dimensional, multimodal neuroimaging data is challenging. Thus, computer-aided machine learning methods for consolidative study have rapidly become extremely popular, and multiple neuroimaging modalities have recently been developed to identify AD. A popular neuroimaging process for examining brain activity in neurodegenerative illnesses is resting-state fMRI (7).

Based on recent research, brain changes associated with AD begin up to two decades before symptoms appear. Due to the high cost and side effects of current medicines, it is essential to focus on enhancing the quality of life or reducing the impact of the disease. To this end, a computer learning model showing significant performance in predicting the disease earlier can help minimize losses (8).

The structure of the study is outlined as follows: Section II provides background on the phases of AD and BOLD data. Section III reviews previous study on fMRI data. Section IV introduces the framework, while Section V shows the results. Section VI discusses the evaluation metrics used, and Section VII compares the findings with previous studies. Finally, Section VIII concludes the study and outlines future research directions.

### 1.1 Motivation and contribution

In recent years, computer-aided design systems have become increasingly important in diagnosing and grading AD, a severe disease affecting many people, particularly the older population. AD causes memory loss and an inability to function in one's environment. The biology of the disease is not yet fully understood, and no cure or medication is currently available to prevent its progress. Early detection is essential for minimizing the impact of the illness and enhancing patients' quality of life. However, classifying AD is challenging due to various constraints involved in fMRI scans, such as low spatial resolution, image artifacts, and motion aftereffects. Despite the low spatial resolution, the abstract and high-level shapes can still provide valuable information for our analysis. With a large amount of data, we have the potential to capture a wide range of variations, which can help improve the robustness and generalization of the model, based on inter subjects. This diversity can also help identify and characterize AD patterns and various sub-types or stages. Addressing these problems at different stages is necessary to develop a robust detection and classification framework for AD.

The primary contributions of this study include:

- To apply techniques using MVPA to consider patterns across multiple variables simultaneously.
- To identify relevant features in order to mitigate the impact of irrelevant or redundant ones by using the LASSO method.
- To propose a framework for detecting AD based on brain signals using hybrid machine learning classifiers.
- To evaluate the results using performance metrics on the public datasets of OASIS and ADNI for improved accuracy rates.

### 1.2 Early diagnosis benefits

Early detection of AD is crucial for several reasons (9):

- Early intervention: It is referred to as the strategies implemented for the early detection of AD. As there is no treatment for AD, the progression of the disease can help to manage the symptoms (10).
- Treatment planning: Early detection of AD allows for the timely implementation of comprehensive treatment plans, including medications, lifestyle changes, and cognitive interventions.
- Clinical trials: Early detection enables individuals to participate in clinical trials for new treatments, which are crucial for advancing our understanding of AD and developing new therapies.
- Learn about the management of AD symptoms.
- Develop a community for assistance.
- Conduct clinical studies to test any recent possible medication (9).

## 2 Background

### 2.1 Phases of AD

**Figure 1:**
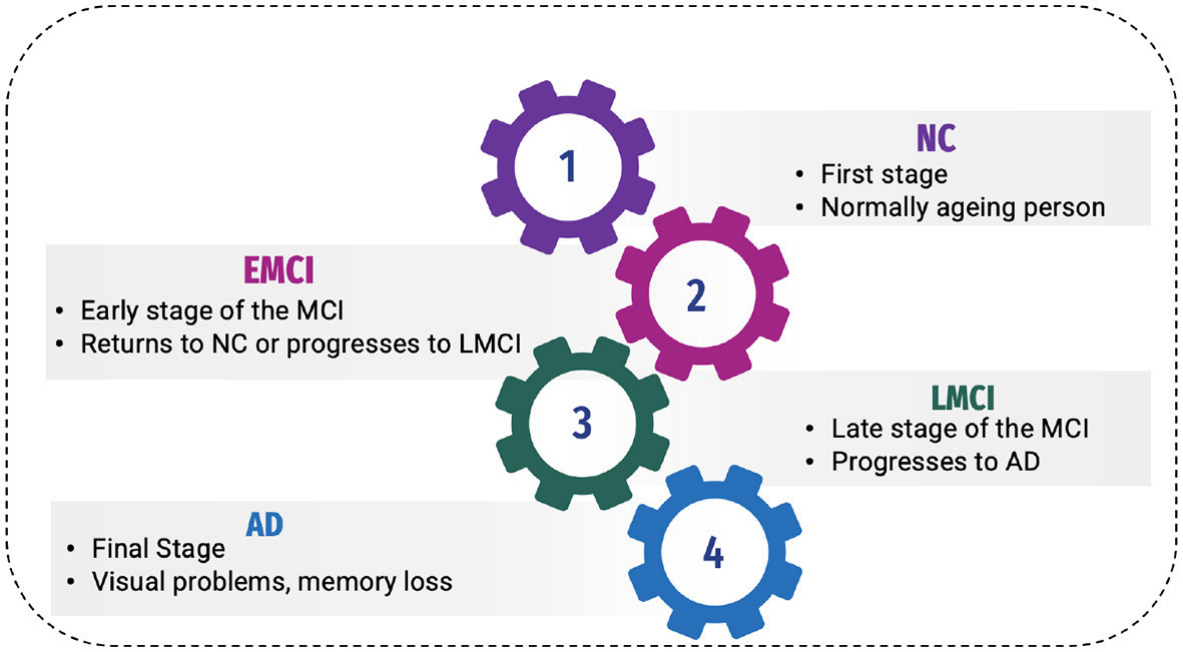
Phases of AD.

AD has been classified into four stages (11), as shown in Figure 1:

#### 2.1.1 Normal control

Normal control is also known as cognitive normal, which is the natural process of cognitive aging. Individuals of 66 years of age healthily, retaining their ability to think, respond and communicate. This is related to the natural aging process (12). They show no symptoms of AD.

#### 2.1.2 Mild Cognitive Impairment or prodromal stage

The intermediate stage between healthy control and AD is referred to as MCI. During this stage, an individual experiences short-term memory loss and difficulty remembering the names of familiar people or objects as a symptom. According to studies, 80% of MCI patients advance to AD after a certain time period of ~5–6 years (12).

Individuals may experience minor abnormalities in cognitive function, but they are insufficiently severe to meet the criteria for the diagnosis of Alzheimer's disease in Early Mild Cognitive Impairment (EMCI stage) (13). Therefore, this stage is generally considered harmless. Not everyone with MCI will develop AD, and some people may even show improvement in their mental abilities. This stage damages the medial temporal lobe of the hippocampus and causes symptoms of short-term memory loss.

More progression is toward another alarming stage, which is Late Mild Cognitive Impairment (LMCI) (13), affecting the lateral and parietal lobes of the brain. Reading difficulties, poor object recognition, difficulty knowing the names of people, and a lack of sense of direction are all symptoms of this stage.

#### 2.1.3 Alzheimer's disease

**Figure 2:**
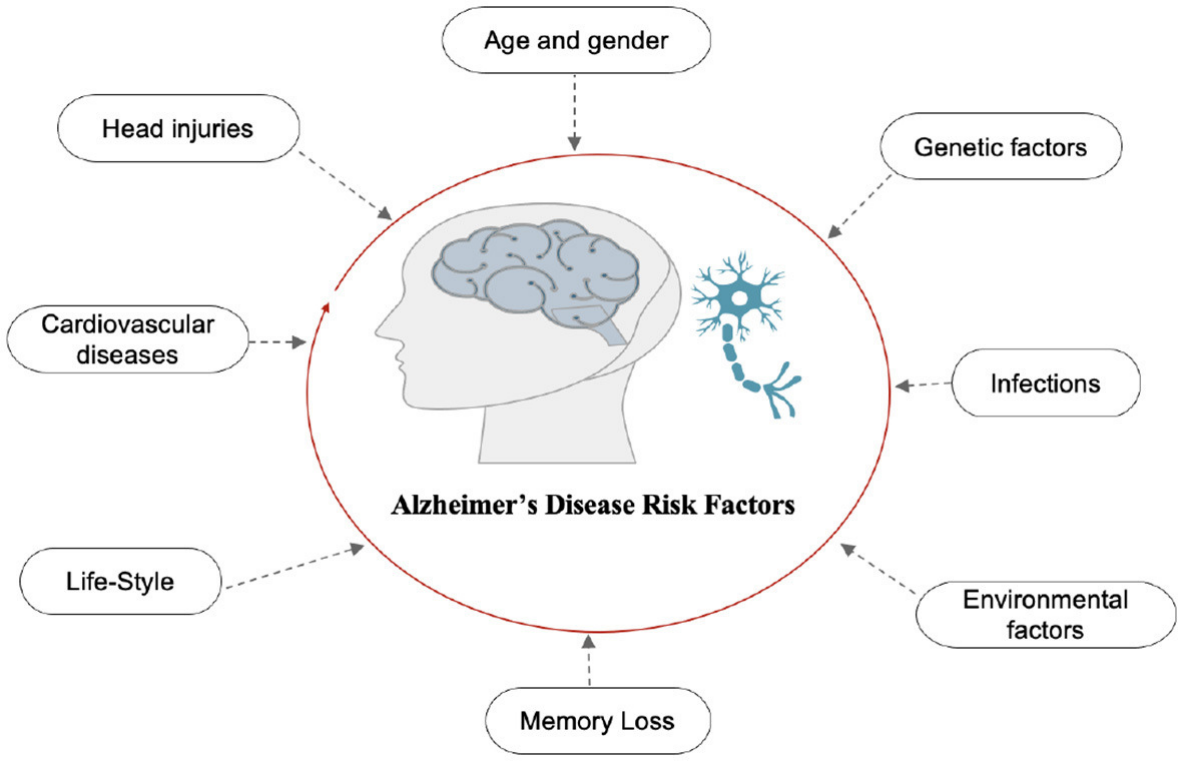
Risk factors of AD.

AD is the final stage of the disease, characterized by severe memory loss, including the names of people and things. This stage is incurable (14). The stage of AD begins in the hippocampus and entorhinal cortex and gradually spreads to other brain sections, affecting the frontal, temporal, and occipital lobes of the brain. Poor judgment, impulsivity, a short attention span, and vision issues are all symptoms of this period. Advancing age, hereditary variables, brain traumas, vascular illnesses, pathogens, and external conditions are among the risk factors contributing to AD development, as shown in Figure 2. What leads to the pathological changes observed in AD remains unclear. While several theories exist, two of the most prominent ones suggest that cholinergic dysfunction and amyloid protein abnormalities may be significant risk factors. However, no widely accepted explanation exists for the underlying mechanisms of AD (15).

### 2.2 Blood Oxygenation Level-Dependent signal

**Figure 3:**
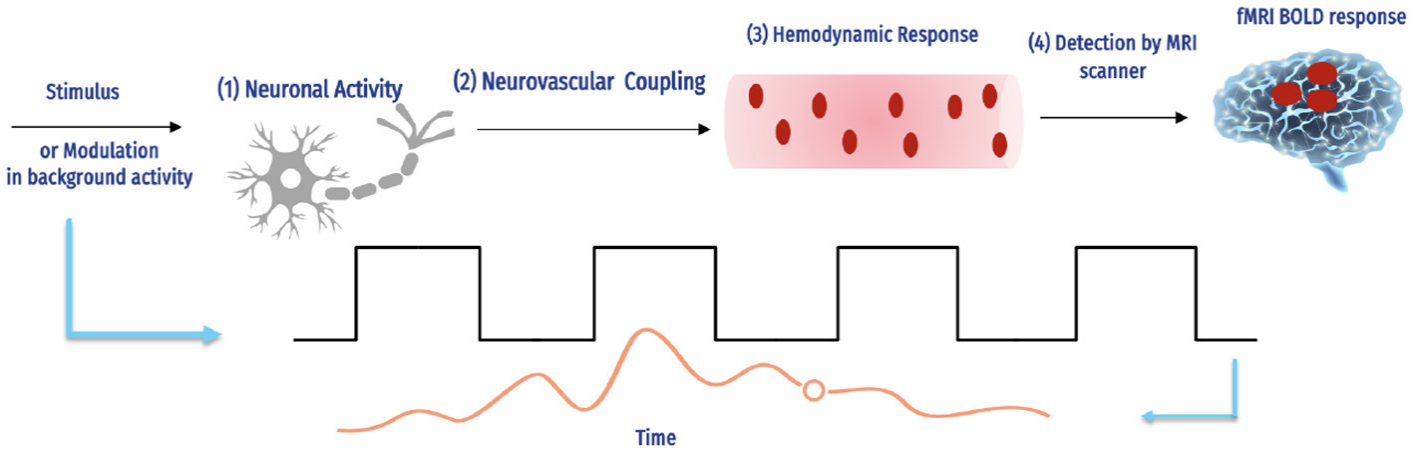
The fMRI BOLD signals and hemodynamic response (16).

Several important factors influence the BOLD signal, as shown in Figure 3.

The complex interaction between neural action and causing a hemodynamic reply, and how an MRI scanner can detect this response. The magnetic field intensity, echo duration, and type of imaging technology used are only a few of the experimental factors in the scanning of fMRI that influence the number of BOLD signals detected by each scanner. For instance, although the hemodynamic response is the same, a 1% BOLD signal throughout an echo of 30 ms is comparable to 2% over an echo period of 60 ms, and the reaction is continuous. Additionally, BOLD imaging is prone to several aberrations, including field inhomogeneities, ghosting, and head motion (17). Determining how accurately the BOLD reply imitates a specific hemodynamic response is challenging due to the number of interacting variables.

The balloon method by Buxton et al. (18) has been developed through extensive research on the type of hemodynamic reply, particularly by Friston et al. (19). As previously mentioned by Buxton et al. (18), the BOLD signal vascular basis is primarily thought to be a relative inequality between rises in blood flow of local cerebral and concomitant (albeit smaller) rises in oxygen digestion, resulting in a brief drop in the deoxyhemoglobin to oxyhemoglobin ratio.

The blood volume, hematocrit, vascular geometry, and oxygenation levels of basal are other physiological variables affecting changes in the deoxyhemoglobin concentration (20, 21). Despite these crucial starting conditions, the hemodynamic response can differ significantly between species and cortical areas. Different facets of the hemodynamic response may alter on various timescales and have various neuronal underpinnings and effects on the signal of BOLD. It is now widely acknowledged that the signal of BOLD also occurs at prominent draining veins, possibly a few centimeters below the neuronally active part, in addition to capillaries. Inferentially, such changes in the signal would be located spatially apart from the stimulated brain tissue.

Consequently, regarding the "brain vs. vein" debate (22), suggest that the density is based on microvascular, which will consistently be less than that of neurons (23), is impeded by massive contributions of vessels, and is more likely to be the aspect restraining the BOLD-based fMRI spatial resolution. The spin-echo fMRI method reduces these vein contributions, making them potentially useful for more precisely tracing the neuronal sources of fMRI BOLD (24, 25). Capillaries have a more significant impact on image intensity (26), with stronger field strengths. Therefore, these two might become more beneficial when used together.

## 3 Literature review

Several studies have developed ideas for systems that could be used to classify AD. This section examines current studies using deep learning (DL) and machine learning (ML) models in systems for diagnosing and detecting AD. Some previous studies on detecting this disease have used standard ML methods (27). Additionally, many neuroimaging studies feature extraction strategies for fMRI signals; for example, Lama and Kwon (28) implemented graph theory to help predict AD at three stages: AD, MC, and NC, with classifications based on the linear support vector machine (SVM) and the regularized extreme learning machine. The Node2vec graph embedding approach converts graph features into feature vectors.

Parmar et al. (29) developed a 3D-CNN that uses rs-fMRI data to predict AD development. By employing unconventional techniques, they extracted patterns from neuroimaging data and found that a simple deep-learning model works well in categorizing AD. The findings of the study suggest a promising future, where fMRI-based biomarkers could assist in the early diagnosis and classification of AD. The study achieved 96.67% accuracy.

Guo and Zhang (30) introduced a distinct network using an autoencoder(AE) to detect natural aging and progression disorders. The network is based on biased neural networks and can easily diagnose AD. The researchers evaluated the system using the fMRI AD dataset and observed that it provides 25% better accuracy than other methods. The study achieved a remarkable 94.6% accuracy. Another study by Alarjani et al. (31) compared machine learning (ML) and deep learning (DL) models for early detection of AD using fMRI data. A 3D convolutional neural network (3D-CNN) extracted features from support vector machine (SVM) for classification. The 3D-CNN achieved 98.3% accuracy, while the SVM achieved 97.5%.

Shahparian et al. (32) developed an ML-based system that detected AD using fMRI images. The system is used to calculate time series for specific anatomical regions using the individual's fMRI data, and the latent low-rank representation method is utilized to extract pertinent features. Based on the acquired characteristics, the SVM classifier determines whether the person is healthy at the onset of the disease or has AD. The proposed method has an accuracy exceeding 97.5%. The problem with vascular dementia (VD) and AD is that both are more frequent. These may cause controversial diagnoses while using classical MRI and clinical methods. Castellazzi et al. (33) different ML algorithms alongside combinations of MRI data are analyzed. AD and VD are two of the most common. Concerning AD and VD, they may demonstrate multiple neurological symptoms that may lead to ambiguous diagnoses when using MRI criteria and conventional clinical. To overcome this problem, a method to classify AD and VD is presented. The system is assessed by three algorithms, such as ANN, SVM, and neuro-fuzzy inference.

Wang and Lim (34) conducted a new assessment approach introduced for individuals with AD and MCI compared with NC individuals, which utilized the zoom-in neural network DL algorithm. By extracting features from the resting-state fMRI dataset obtained from the ADNI, the algorithm could detect the implicated regions during AD by utilizing the automated anatomical labeling (AAL) Atlas. The study found that the ZNN obtained good results of 97.7, 84.8, and 72.7% accuracy for distinguishing AD from NC and MCI, NC from MCI and AD, and MCI from NC and AD, respectively. This was achieved using seven discriminative ROIs in the AAL-90.

Data optimization is indeed a complex task in the field of neuroimaging. However, Zamani et al. (35) proposed an interesting approach integrating artificial neural network (ANN) with evolutionary algorithms to optimize the neuroimaging data with multiple parameters. Using the rs-fMRI data based on the resting state, they measured the FC and computed 1,155 parameters. They tested the system using the ADNI dataset and achieved 94% accuracy.

To achieve AD discrimination at various stages, Nguyen et al. (36) suggested a voxel-wise discriminative system for multi-measuring rs-fMRI and combining hybrid MVPA and extreme learning machine (ELMs) and applied it to two different datasets. Jiao et al. (37) proposed a method focusing on the multi-scale combination of features. This approach utilizes global static features, moment features, and more refined features extracted from networks that are static, dynamic, and high-order functional. Subsequently, SVM was used to classify EMCI versus NC. Lu et al. (38) developed a system categorizing AD, MCI, and CN of fMRI data using FC throughout the brain rather than feature selection. They then used an ELM to classify binary stages. Unfortunately, this framework is only appropriate for a small dataset.

Yang et al. (39) extensively applied the brain function network to classify AD biomarkers 240 in the MCI stage. They used multiple time points of rs-fMRI data by combining the fused sparse network model based on centralized learning that is parameter-free. The essential features selected by the similarity network fusion method were then used to classify them using SVM. In addition, Chan et al. (40) proposed approach for AD uses a graph neural network (GNN) on MRI and fMRI scans. It encodes scans into brain graphs, clusters representations learned by the GNN to identify disease subtypes, and constructs population graphs for final decision-making. This approach outperforms existing methods, identifying three AD subtypes and revealing unique biomarkers, such as left cuneus and left isthmus cingulate cortex degeneration.

Lama et al. (41) constructed the brain network using Pearson's correlation-based FC of fMRI data. The brain network's graph features were transformed into feature vectors using the Node2vec graph embedding technique. Furthermore, they selected features using various approaches, which they then applied to classifiers: single-layered extreme learning and multi-layered ELM. Koluragi et al. (42) combined SVM and EfficientNetB0 to improve the performance. The integrated approach outperformed individuals, leveraging EfficientNetB0's efficient resource utilization and balance.

In earlier research, rs-fMRI used a mono-band frequency range and focused on low-order neurodynamics. Thus, high-order neurodynamics were deliberately excluded. To address these issues, Sethuraman et al. (43) proposed an automated system to detect AD using rs-fMRI. The system constructs a high-order neurodynamic functional network using different levels of rs-fMRI time-series data, such as slow4 and slow5, and the full-band ranges from 0.027 to 0.08 Hz, 0.01 to 0.027 Hz, and 0.01 to 0.08 Hz. SVM and k-nearest neighbor (KNN) were used for ML, and AlexNet and Inception were implemented to classify various stages of AD. The system achieved 96.61% accuracy in differentiating between AD and NC. Begum and Selvaraj (44) used deep CNN (DCNN) and 3D densely connected convolutional neural network algorithms to diagnose AD and perform feature analysis on fMRI data.

To enhance early detection (45), the effectiveness of Extreme Learning Machines (ELMs) was assessed alongside fMRI-based FC metrics. The non-linear methods such as MIC and eMIC were applied as classification features leads to robust outcomes. The study achieved a 95% accuracy rate in distinguishing between AD and NC using these methods. The study conducted by Penalba-Sánchez et al. (46) investigated the dynamic and static FC of resting-state fMRI using various methods across 116 ROIs for four participant groups. Additionally, they utilized graph theory metrics to investigate network segregation and integration. The results showed that the EMCI group had a longer typical path length and lower degree compared with the healthy control (HC) group.

### 3.1 Important of gap

MVPA techniques can enhance the ability to detect significant changes in the activity of the brain that may not be noticeable with traditional univariate methods. This is particularly important in AD, where early detection of subtle changes can be crucial for timely intervention. Additionally, MVPA allows a more detailed understanding of how different brain regions interact and contribute to cognitive processes. This can provide valuable insights into the underlying mechanisms of AD and other neurological disorders.

## 4 Proposed framework

**Figure 4:**
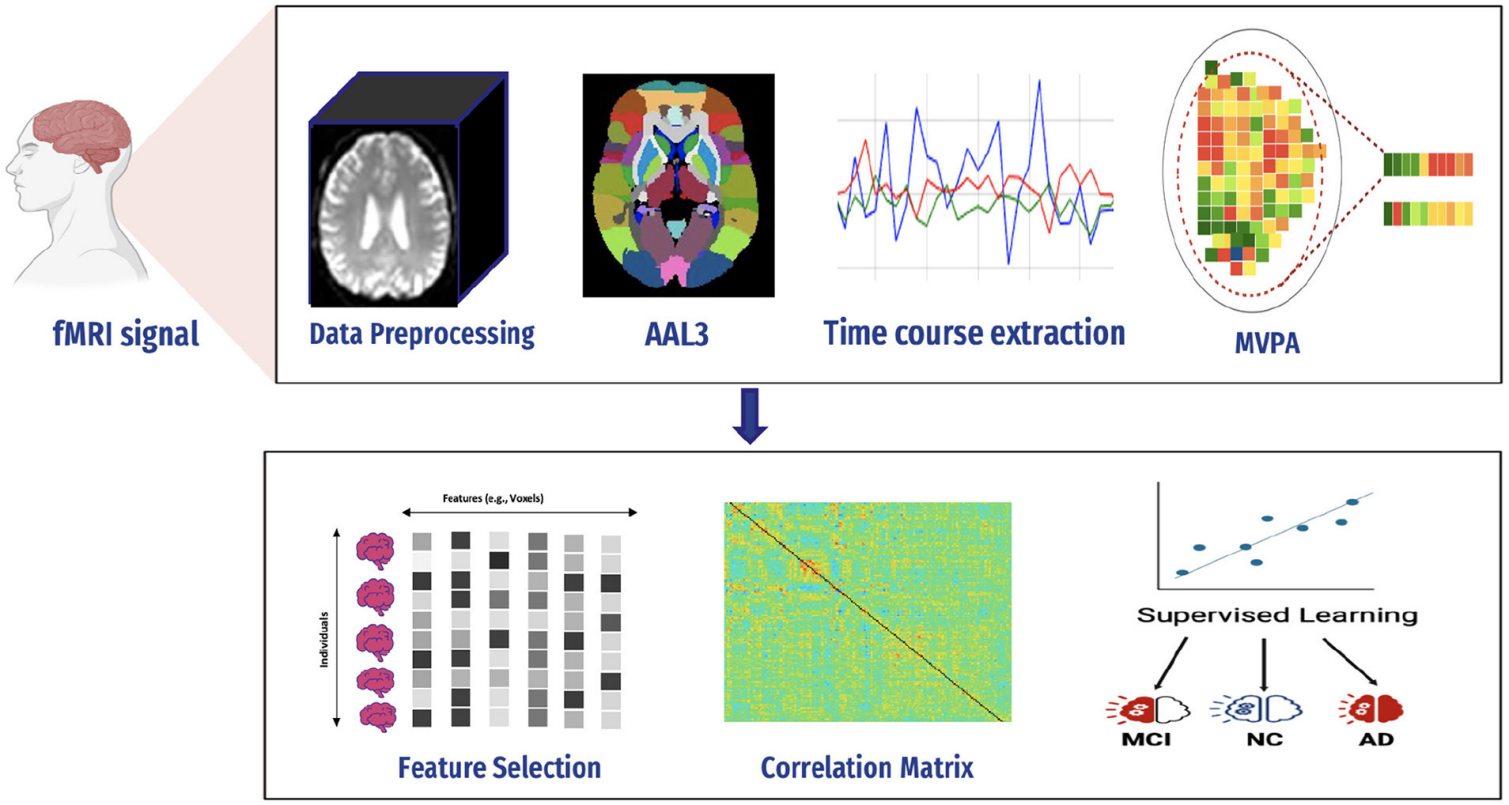
Proposed framework.

AD is a serious health condition affecting many people, particularly the older population worldwide. It is a debilitating illness causing memory loss and impairing one's ability to interact with their surroundings. Early detection is crucial in mitigating the effects of Alzheimer's disease and improving the quality of life. Recognizing the disease at its onset enables the reduction of its impact on patients. We constructed a predictive framework to detect AD at an early stage based on human brain imaging techniques: fMRI. Figure 4 presents a summary of the proposed framework. It includes the following steps: (1) data collection (i.e., fMRI), (2) preprocessing of fMRI data to avoid articles (i.e., noisy), (3) computing FC through MVPA, (4) extracting time series of fMRI data, (5) computing correlation matrices for each stage, (6) feature selection to select relevant features (i.e., voxel), (7) supervised learning, and (8) evaluation and analysis.

### 4.1 fMRI signal preprocessing

Since medical images are complex and difficult to extract features, various techniques must be used to process images in the dataset.

A flexible preprocessing pipeline is used to prepare functional and structured data, including realignment, slice timing correction (STC), normalization to MNI space, and smoothing (47). For realignment, we utilized the SPM realignment unwarping procedure suggested by Andersson et al. (48). Then, scans are co-registered based on a reference image, such as the first scan of the first session. For this, a least square technique and a transformation of a 6-parameter (rigid body) are utilized, as presented in the study by Friston et al. (49). After that, the interpolation of the B-spline was resampled to reduce the effects of motive and magnetic artifacts.

**Figure 5:**
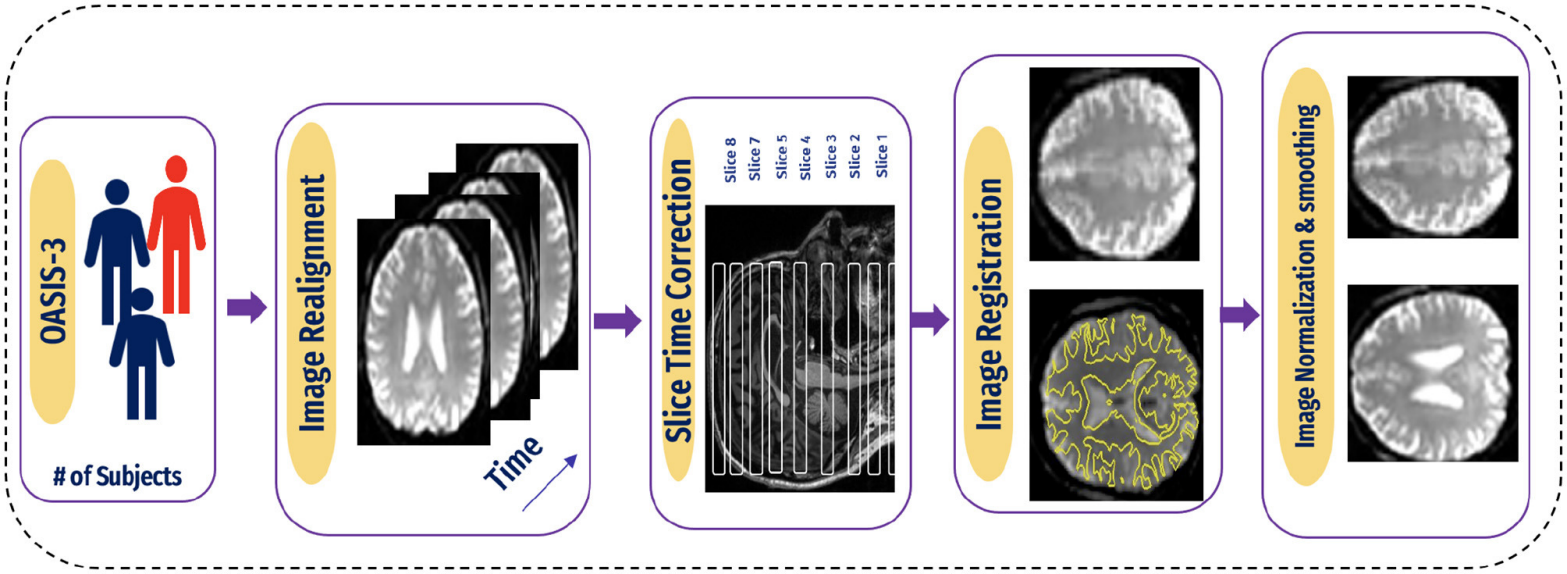
Pipeline for preprocessing of BOLD data.

Temporal misalignment and methods were applied to identify scans. A reference BOLD image was developed by applying the mean to the scans, and the outliers were excluded. The SPM unified normalization algorithm is used to perform the normalization and obtain the standard MNI space (50, 51), with the probability map template based on default IXI-549 tissue, as resampled to 2 mm isotropic voxels. Finally, the spatial convolution of the data was performed with the help of a Gaussian kernel of 6 mm full-width at half-maximum (FWHM) for smoothing (see Figure 5 for an illustration).

### 4.2 Functional connectivity

An essential application of fMRI studies is brain network mapping in AD patients and between the brain network mapping routes. At rest, the default mode network is among the most exciting networks (52). DMN relates to knowing previous events, imagining future events, self-relevant mental processing, and checking external information (53). Alterations in DMN functional activity have been linked to neurological disorders (54–56). Most studies show decreased FC in the DMN. In a study by Koch et al. (57), the power of the DMN in rs-fMRI was examined to differentiate between three groups: CN individuals, MCI, and patients with AD. Moreover, this can be constructed using numerous imaging technologies [for example, EEG/magnetoencephalography [MEG] and structural, diffusion, and functional MRI]. Ways to analyze FC include UNIVAR and MVPA.

#### 4.2.1 Univariate analysis

**Figure 6:**
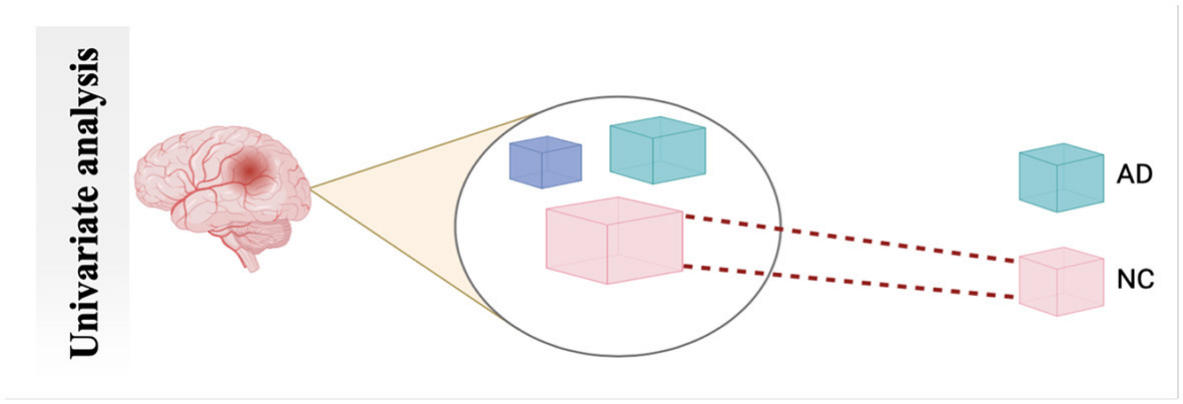
Schematic diagram of univariate analysis.

UNIVAR is a method used to analyze fMRI data. UNIVAR assesses the individual voxel neural activation or the average voxel activation of the brain. Thus, it is used for the localization of brain regions participating in processing specific stimuli such as face versus object. The conclusion about the brain regions participating in cognitive processes is also drawn from the study by Haynes and Rees (58). A general linear model is employed on each voxel, which is why it is called univariate (59). FCA characterizes communication between various brain regions during a task or rest. It also measures the relationship strength between the BOLD signal of the time series (60), as shown in Figure 6.


(1) $$\begin{array}{l} \forall x,y\;r_{n}\left(x,y\right)=g_{n}*b\left(x,y\right)+\epsilon_{n}\left(x,y\right).\sigma \left(x,y\right) \end{array}$$

Null hypothesis *C*.*b*(*x, y*) = 0

Here in Equation 1, n refers to the number of subjects in a study, and x and y are the voxel pair. The characterizing FC of these two voxels can be considered as *r*_*n*_(*x, y*), where *g*_*n*_ is referred to as the vector of a predictor of each subject n. The unknown regression coefficient of an unknown vector is b(x,y), while ϵ_*n*_ (x,y) and σ (x,y) are error term and inter-subject variance, respectively. A null hypothesis can be formed using *C*.*b*(*x, y*) = 0.

Many studies used UNIVAR, such as in the study by Moeller et al. (61), to identify the region's dynamic activity close to the expected waveform. In another method Bu et al. (62), the authors examined the UNIVAR and MVPA overlap.

#### 4.2.2 MultiVoxel (or Multivariate) Pattern Analysis

**Figure 7:**
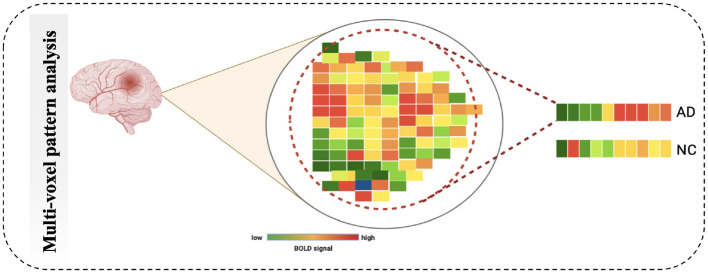
Schematic diagram of Multi-Voxel Pattern Analysis.

Multivariate Pattern Analysis MVPA is the most used technique for analyzing functional data. In this study, the spatial pattern of neural activation across various voxels is considered (e.g., voxels in fMRI or channels in MEG/EEG). It also assesses whether it has information related to the task (63). It is called multivariate because it is based on analyzing a set of voxels rather than single voxel modeling (64). The similarities of such patterns can also be investigated by the activation of these patterns, such as by viewing a scene vs. a face, Norman et al. (65), as shown in Figure 7. The MVPA can be mathematically defined as follows (Equation 2).


(2) $$\begin{array}{l} \forall x\;r_{n}\left(x\right)=g_{n}*B\left(x\right)+\epsilon_{n}\left(x\right)*\sum \left(x\right) \end{array}$$

Null hypothesis *C*.*B*(*x*).*P*(*x*) = 0

While *r*_*n*_ (x) refers to connectivity value whole map, unknown predictor of regression coefficients is denoted by B(x). ϵ_*n*_ (x) refers to residual error. ∑(*x*) is denoted as voxel-by-voxel matrix of positive definite. While C denotes between subject, P(x) represents contrast matrix of between-voxels. There are many studies that used MVPA such as in the study by Yoon et al. (66), it used validate impairment hypothesis in schizophrenia-distributed representations. In another method, Lee et al. (67) conducted hypothesiss by using MVPA to check that based on the brain prediction, the efficiency of models has variations across the stimuli types.

### 4.3 Region of interest

**Figure 8:**
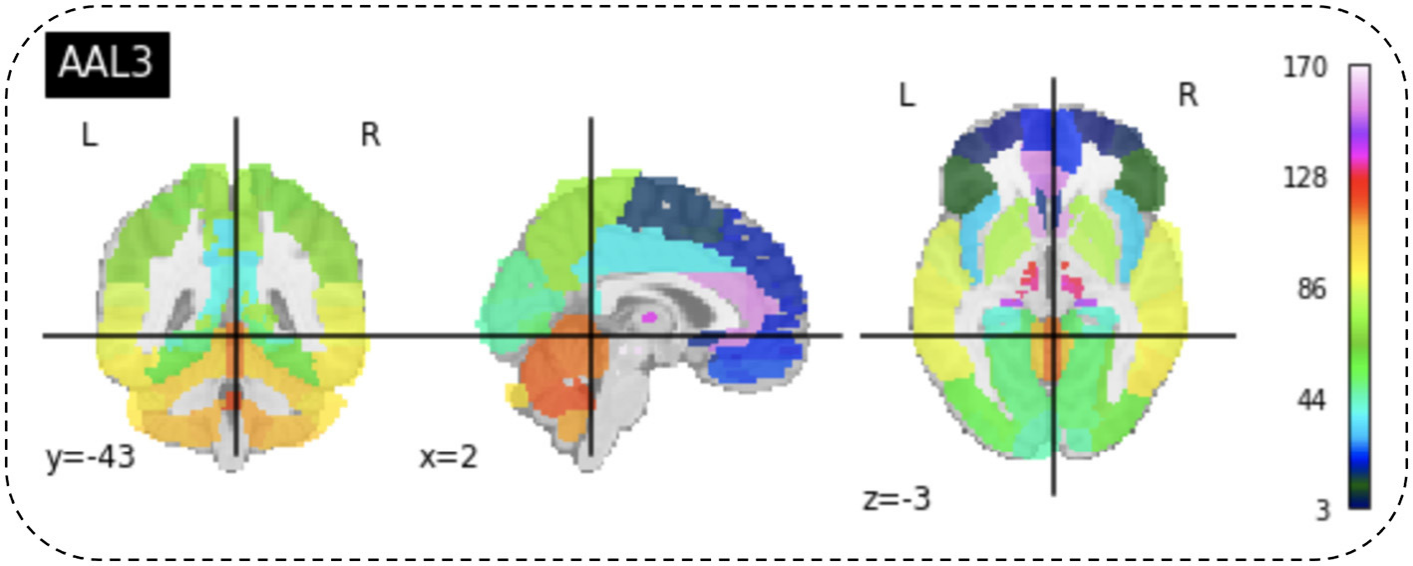
View for the AAL3 template.

**Figure 9:**
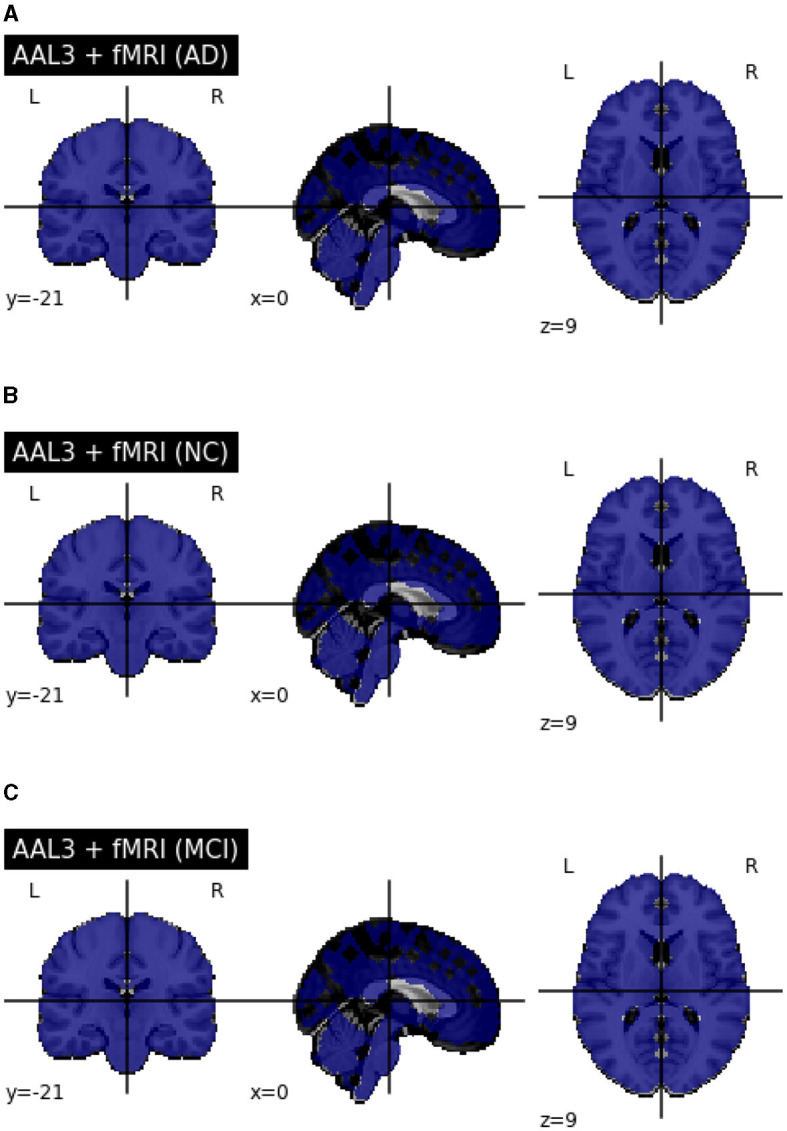
**(A)** Performed mask and functional of AD. **(B)** Performed mask and functional of MCI. **(C)** Performed mask and functional of NC.

After preprocessing BOLD fMRI data, we can extract features from the fMRI data depending on the atlas. Automated Anatomical Labeling (AAL) atlas is a tool used in neuroimaging that provides a pre-defined anatomical division of the human brain. This tool is widely used in neuroscience research, particularly in functional and structural brain imaging studies, such as fMRI and PET. The AAL atlas helps researchers to identify and label specific brain regions in their neuroimaging data. The human brain is divided into anatomical regions, each with a specific label in the AAL atlas. AAL atlas provides standardized three-dimensional coordinates for each region, which researchers can use to locate and precisely label brain imaging data areas. The AAL atlas performs various analyzes, including region-of-interest (ROI) studies in functional brain imaging, to map brain activity during specific tasks or resting-state conditions (68). Few types of the AAL atlas are as follows: AAL1 (69), AAL2 (70), Chinese AAL (71), AAL3 (68). Dealing with high-dimensional and small sample datasets such as fMRI data is challenging when it comes to classification and modeling. To address this issue, the AAL template is utilized in this study to calculate the functional link matrix after processing the original image. In Figure 8, the AAL3 used to perform feature extraction to identify relevant brain regions or patterns for the fMRI. AAL3 includes 170 regions, masking objects with an atlas to extract time series within each ROI (see Figure 9).

### 4.4 Compute connectivity

**Figure 10:**
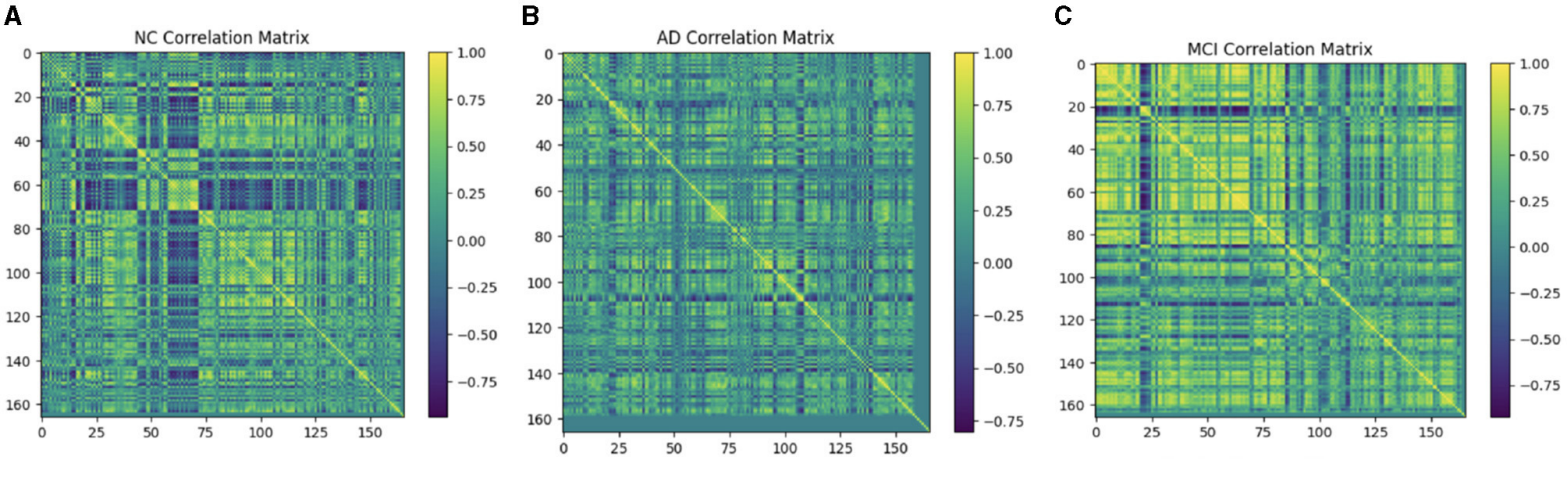
Functional connection matrix and brain network visualization for each stage (AD, MCI, and NC). **(A)** Functional connectivity NC. **(B)** Functional connectivity AD. **(C)** Functional connectivity MCI.

**Figure 11:**
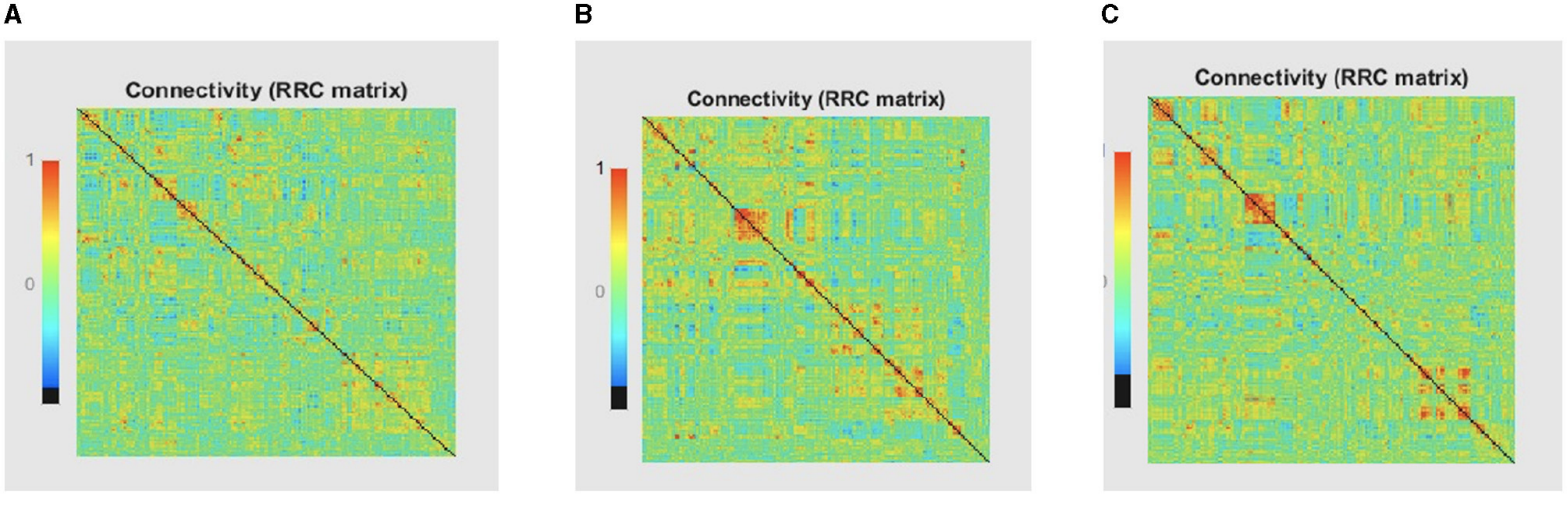
Functional connection matrix and brain network visualization for each stage (AD, MCI, and NC) in ADNI dataset. **(A)** Functional connectivity NC. **(B)** Functional connectivity AD. **(C)** Functional connectivity MCI.

Multiple techniques are available to calculate the FC of fMRI. These techniques include connectivity maps of seed-to-voxel, ROI-to-ROI connectivity matrices, independent component analysis, and multivariate pattern analysis (MVPA). This study proposes FC using MVPA to analyze individual voxel resolution in the brain-wide connectome. This approach uses the MVPA methods to overcome the challenges of brain-wide connectome analysis. MVPA was applied to a 4D BOLD dataset to compute the correlation matrix between voxel time series within each ROI and remove relevant voxels based on their correlation with other voxels. These analyzes calculate a series of associated connectivity patterns and spatial maps that illustrate the voxel connectivity to the rest of the brain. Based on the provided fMRI time-series data, the calculated correlation matrix will then contain correlation values between ROI pairs. The FC matrix is displayed using the AAL3 template, which includes 166 brain regions, resulting in a connectivity matrix of 166 X 166. The correlation matrix ranges from 0 to 1, with 0 indicating no correlation and 1 indicating a high degree of correlation. The matrix is shown in Figures 10, 11.

### 4.5 Feature selection

#### 4.5.1 LASSO

Suppose we have a data $\left(x^{i},Y_{i}\right)$, i = 1, 2, … , N, where *x*^*i*^ = ${\left(X_{il},Y_{ip}\right)}^{T}$ refers to the variables used for prediction, *y*_*i*_ refers to the response. In the usual setup of regression, we suppose that either all observation is independent or *y*_*i*_s are independently conditionally of the given *y*_*ij*_s we can suppose that *X*_*ij*_ referred to as standardized $\underset{\;}{\overset{\;}{\sum_{i}}}x_{ij/N}$ = 0, $\underset{\;}{\overset{\;}{\sum_{i}}}{x^{2}}_{ij/N}$ Suppose $\hat{\beta }={\left({\hat{\beta }}_{1},\ldots ,{\hat{\beta }}_{p}\right)}^{T}$, and the lasso estimate $\left(\hat{\alpha },\hat{\beta }\right)$

Here, *t*≥0 is referred to as a parameter for tuning. For all t, the solution for a is $\hat{\alpha }=ŷ$. We can consider without losing the generality that $\hat{\alpha }=0$ which omit α. The solution of the above equation is a problem of quadratic programming having linear constraints of inequality.

The amount of shrinkage is controlled by the parameter *t*≥0. It is applied for estimation. Suppose ${\hat{\beta }}_{j}$ refers to the estimates of full least squares. Let $\sum |{\hat{\beta }}_{j}|$, then the shrinkage will occur due to *t* < 0. This shrinkage will occur in the solutions toward 0. There are some coefficients and value of these coefficients will be 0. If *t* = *t*_0_/2, then the affect will be same as searching the best subset having a size of *p*/2. It is not necessary that the matrix of design will be of full rank.

The motivation behind the Lasso is from a proposal by Breiman, and it can be defined as Equations 3 and 4.


(3) $$\begin{array}{r} \left(\hat{\alpha },\hat{\beta }\right)=\arg\min\left\{\overset{N}{\underset{i=1}{\sum }}{\left(y_{i}-\alpha -\underset{j}{\sum }\beta_{j}x_{ij}\right)}^{2}\right\}\\ \text{subject to}\underset{j}{\sum }|\beta_{j}|\leq t. \end{array}$$

(4) $$\begin{array}{l} \overset{N}{\underset{i=1}{\sum }}{\left(y_{i}-\alpha -\underset{j}{\sum }c_{j}{\hat{\beta }}_{j}^{{}^{\circ}}x_{ij}\right)}^{2}\text{subject to }c_{j}\geq 0,\;\sum c_{j}\leq t \end{array}$$

**Figure 12:**
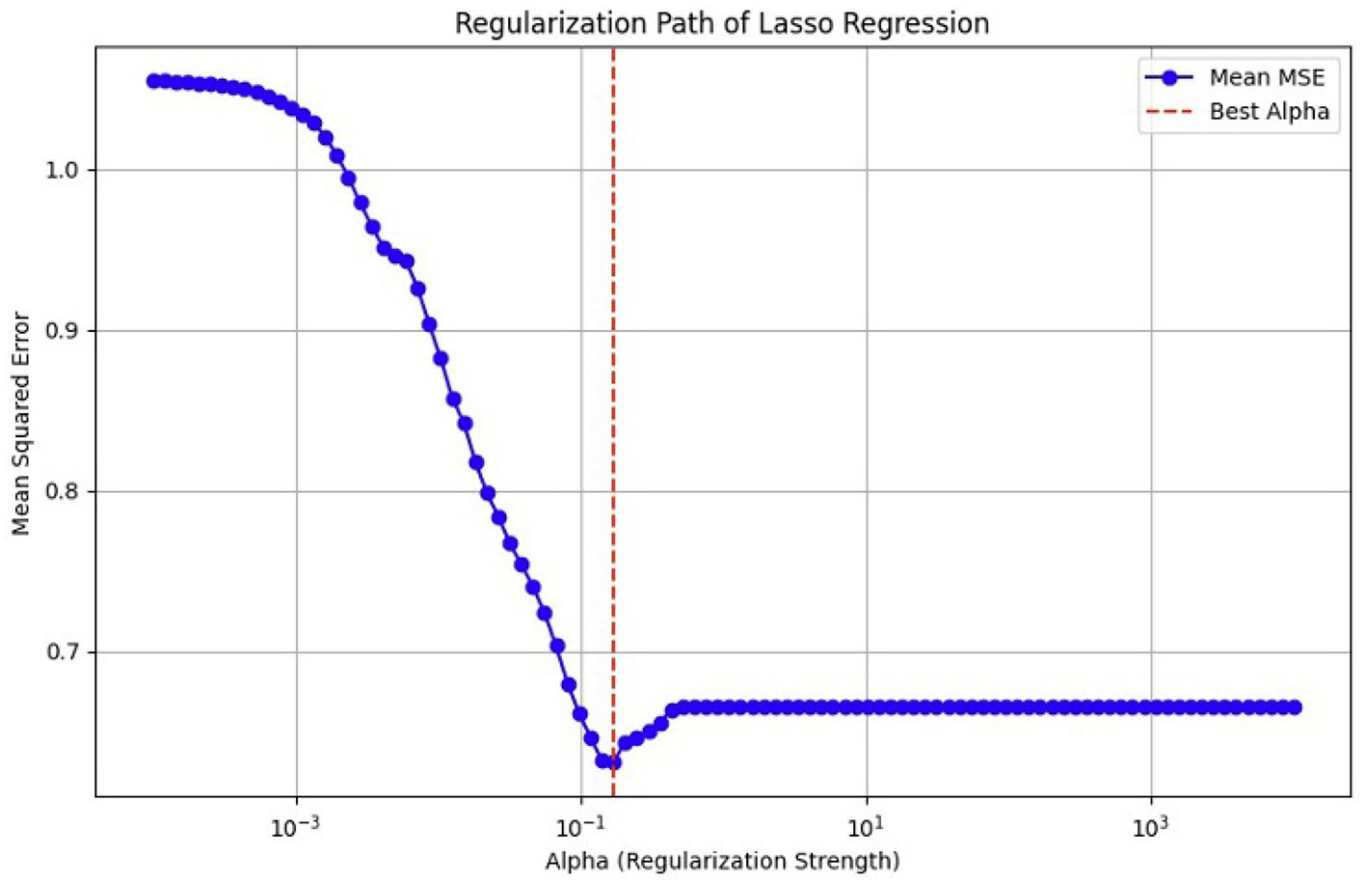
MSE of the LASSO fit, cross-validated with a parameter lambda (λ), for the OASIS dataset.

**Figure 13:**
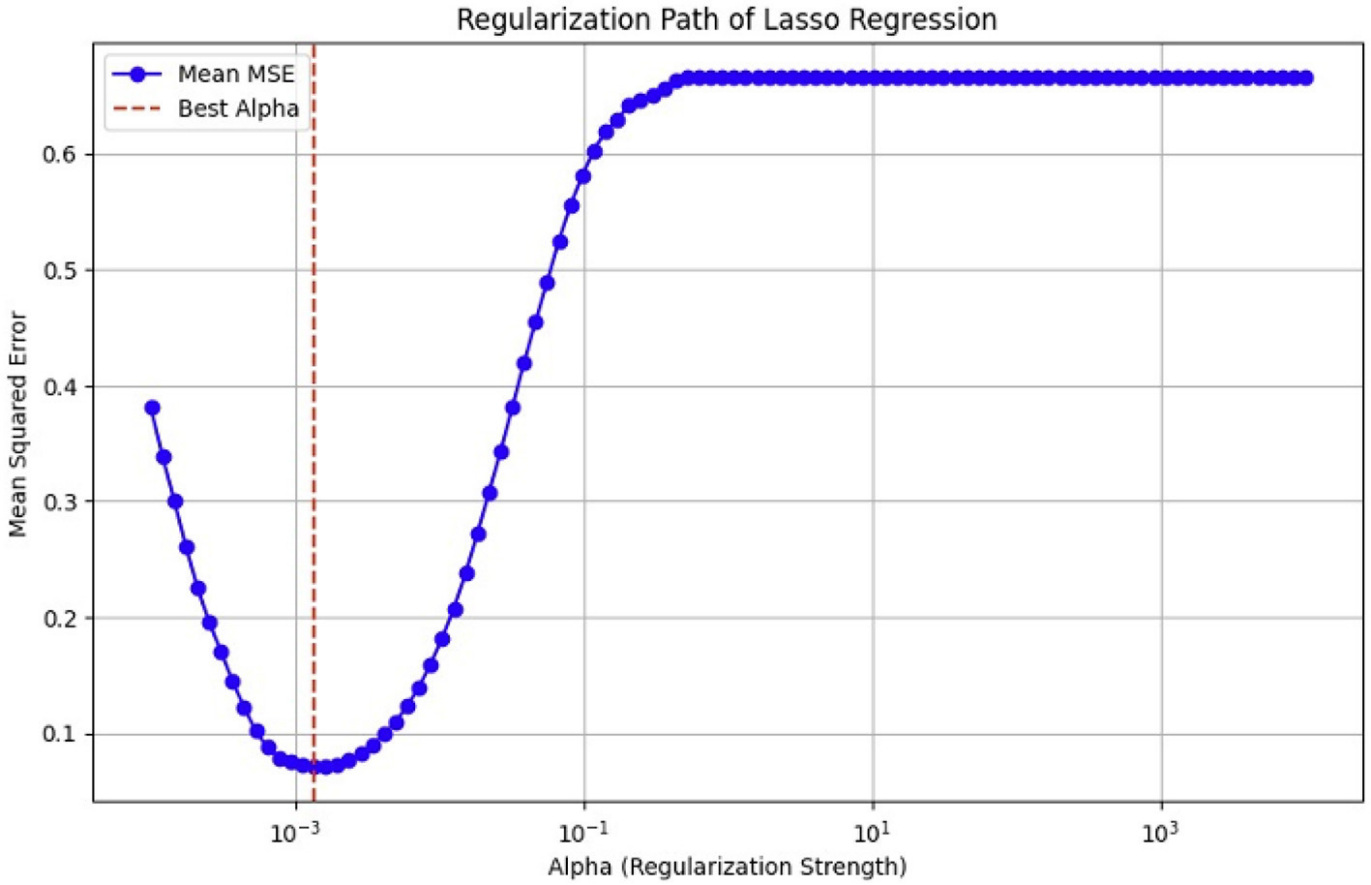
Mean squared error (MSE) of the LASSO fit, cross-validated with a parameter lambda (λ), for the ADNI dataset.

As previously mentioned, fMRI data are high-dimensional, with many voxels (3D pixels) representing regions of the brain. In this context, LASSO helps select a subset of these most relevant voxels for a particular analysis. Lasso is used as a regularization technique in linear regression methods. It adds a penalty term to the sum of squared errors, encouraging sparsity in the resulting model. It promotes the selection of a subset of features (voxels or ROI) while setting others to zero. Identifying relevant voxels or ROIs: Lasso regression is employed to identify relevant features (relevant brain regions or voxels). The Lasso coefficients provide information about the importance of each feature by setting a suitable penalty parameter (alpha = 0.01). Features with non-zero coefficients are considered relevant, and those with coefficients set to zero are effectively excluded from the model (72). We selected the λ value that minimized the cross-validated mean squared error (MSE), as shown in Figures 12, 13.

### 4.6 Machine learning

Machine learning (ML) is among the most efficient and robust tools that have entered the medical imaging domain in the last few years. The recent advances in this field have enabled intelligent algorithms capable of assisting human experts in making wise decisions. Data are prepared in various directions, such as single and hybrid models, to classify the disease by organizing the time series of relevant voxel(s) into a matrix and labeling samples as AD, MCI, or NC based on their task condition or behavioral response. Table 1 presents the hyper-parameters of ML.

**Table 1:** Tuning for machine learning models.

**Model**	**Hyperparameters**
SVM	kernel = "sigmoid", C = 0.2, random state = 300
AdaBoost	Default
KNN	n_neighbors = 300, weights = "uniform", *p* = 2
HML	voting = "hard"

#### 4.6.1 Single model

##### 4.6.1.1 Support vector machine

Support vector machine (SVM) is among the most common classification and regression analysis algorithms. They use patterns found through data analysis and pattern recognition to predict newly collected data. The SVM classifies data into different classes by creating a hyperplane. The nearest points from each class are kept as far apart as feasible by the hyperplane, which is selected to optimize the margin between the two classes (73).

##### 4.6.1.2 K-Nearest Neighbor

K-Nearest Neighbor (KNN) is an important ML model based on supervised learning. The approach assumes similarity between new and existing subjects. Subsequently, it places the latest subject in the group, which is mostly similar to the existing categories, such as AD, MCI, and NC. The KNN algorithm saves all existing data and generates new subjects based on similarity. Once a new subject is developed, the KNN method instantly categorizes it into a suitable category. Notably, KNN is a non-parametric technique, so no assumptions about original data are made. During the training phase, the KNN algorithm stores the dataset and classifies new subjects into a category similar to the old data (74).

##### 4.6.1.3 AdaBoost

AdaBoost classification involves training of numerous weak classifiers on the same training set to create a robust classifier. The weak classifier is a stump of the tree. The models then decide which prediction is the best; however, this technique depends on the weak classifier. It would increase the accuracy if used along with another algorithm (75).

#### 4.6.2 Hybrid approach

**Figure 14:**
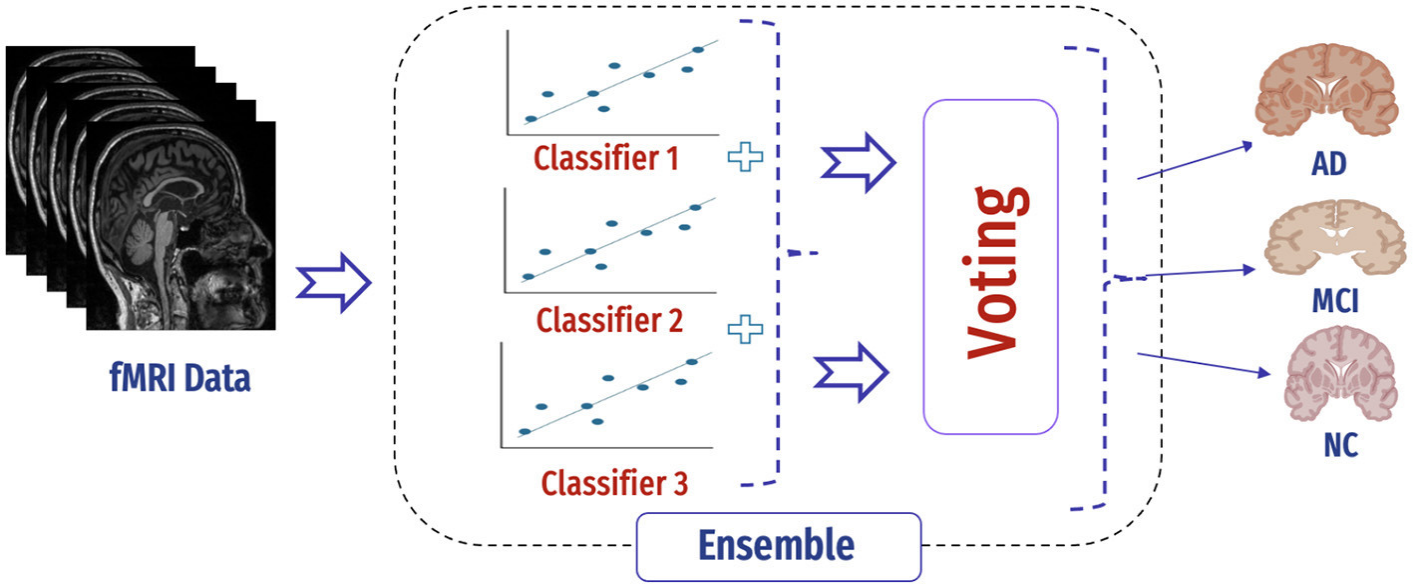
Mechanism of ensemble.

In our case, the ensemble classifier combined the predictions of three base classifiers, namely, SVM, AdaBoost, and KNN classifiers by voting (Figure 14). In soft voting, each data point in fMRI collects the probability estimates (class probabilities) from each of the individual classifiers in the ensemble. The average or weighted average of these probabilities is computed for each class to make a final decision for that data point. The predicted class label for each data point is determined by selecting the class with the highest average probability.

## 5 Results and analysis

### 5.1 Datasets

#### 5.1.1 Open Access Series of Imaging Studies

**Figure 15:**
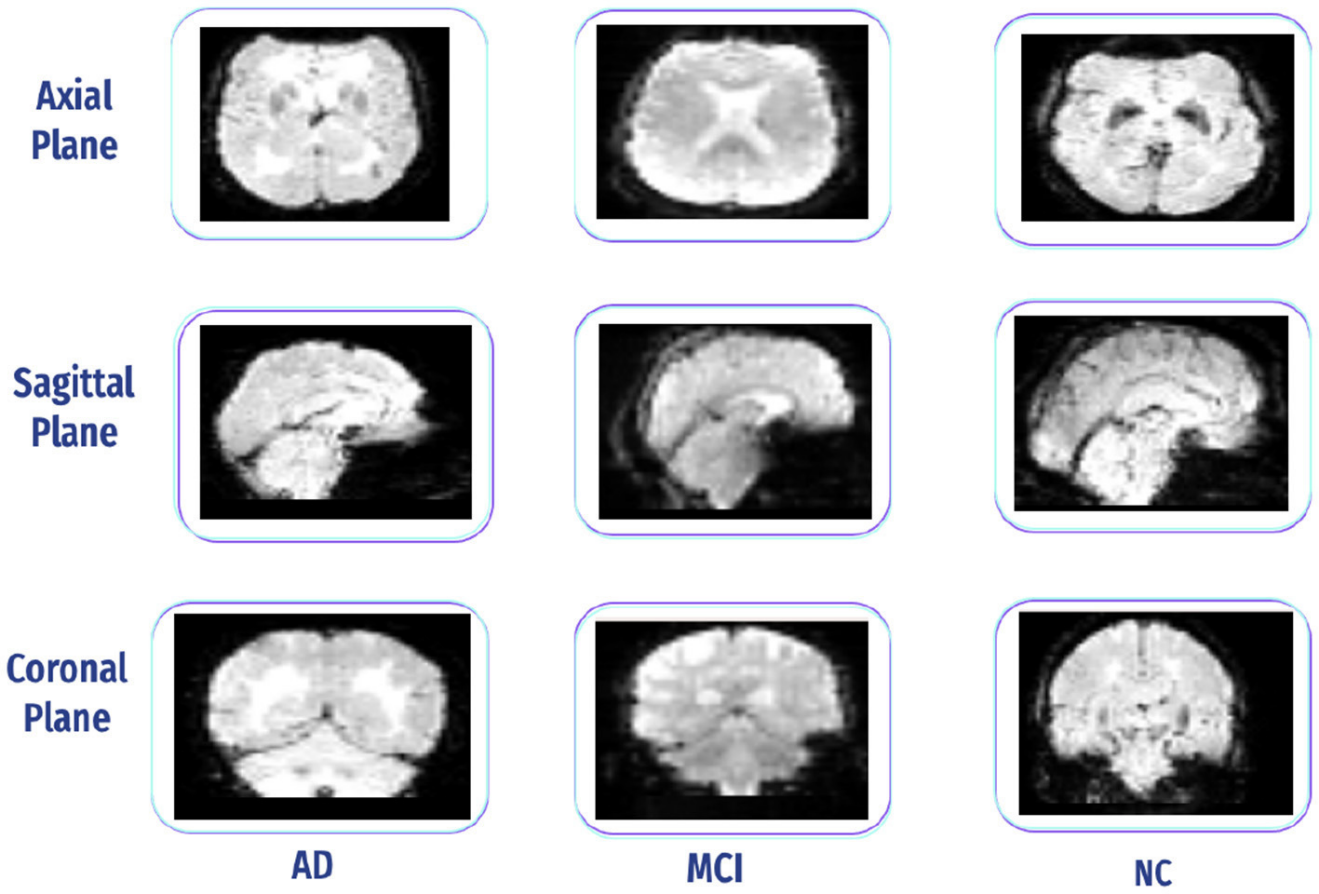
Three-plane view for (AD, CN, and MCI) from the OASIS dataset.

A free-access data set was retrieved from the OASIS dataset. It has three versions: OASIS-I, OASIS-II, and OASIS-III. OASIS-III is a longitudinal neuroimaging, biomarker, cognitive, and clinical dataset for normal aging and AD with ages varying between 42 and 95 years, including 1,379 subjects (male/female) and 2,842 MRI sessions, which include T1w, T2w, and resting-state BOLD (rs-BOLD). In our case, We used rs-BOLD data, typically acquired as a sequence of 3D brain volumes, with each volume representing a snapshot of brain activity, including all data for the mild cognitive impairment (MCI) stage, with all slices. We balanced the selection by choosing approximately the same number of samples for AD and normal cognition (NC) classes to prevent bias. Figure 15 presents all three plane views of fMRI data. It has the following requirements: Each stage has several subjects (males and females), including functional and structural data (i.e., T1 W), and each subject has an array of size 64 * 64 * 36 * 164, representing height * width * number of horizontal slices * number of data points. The data are captured using 3.0 T (Tesla) scanners with a slice thickness of 2.4 mm, and the flip angle is 80 degrees (76). Table 2 displays the subjects' demographic information.

**Table 2:** Subject cohort of fMRI (OASIS dataset).

	**AD**	**MCI**	**NC**
Type of image	DICOM^a^		
Number of subjects	101	95	102
Total of slice	16,564	15,580	16,728
Sex (M/F)	72/29	40/55	60/42
Clinical dementia rating (CDR)	0.5	0	1
Flip angle	77°		
Voxel size in fMRI	3*x*3*x*3*mm*^3^		
TR/TE	2 s/25 ms		
Width	64	64	64
Height	64	64	64
Acquisition scanner	3.0 T (Tesla)		

^a^ Digital Imaging and Communications in Medicine.

#### 5.1.2 AD Neuroimaging Initiative

**Figure 16:**
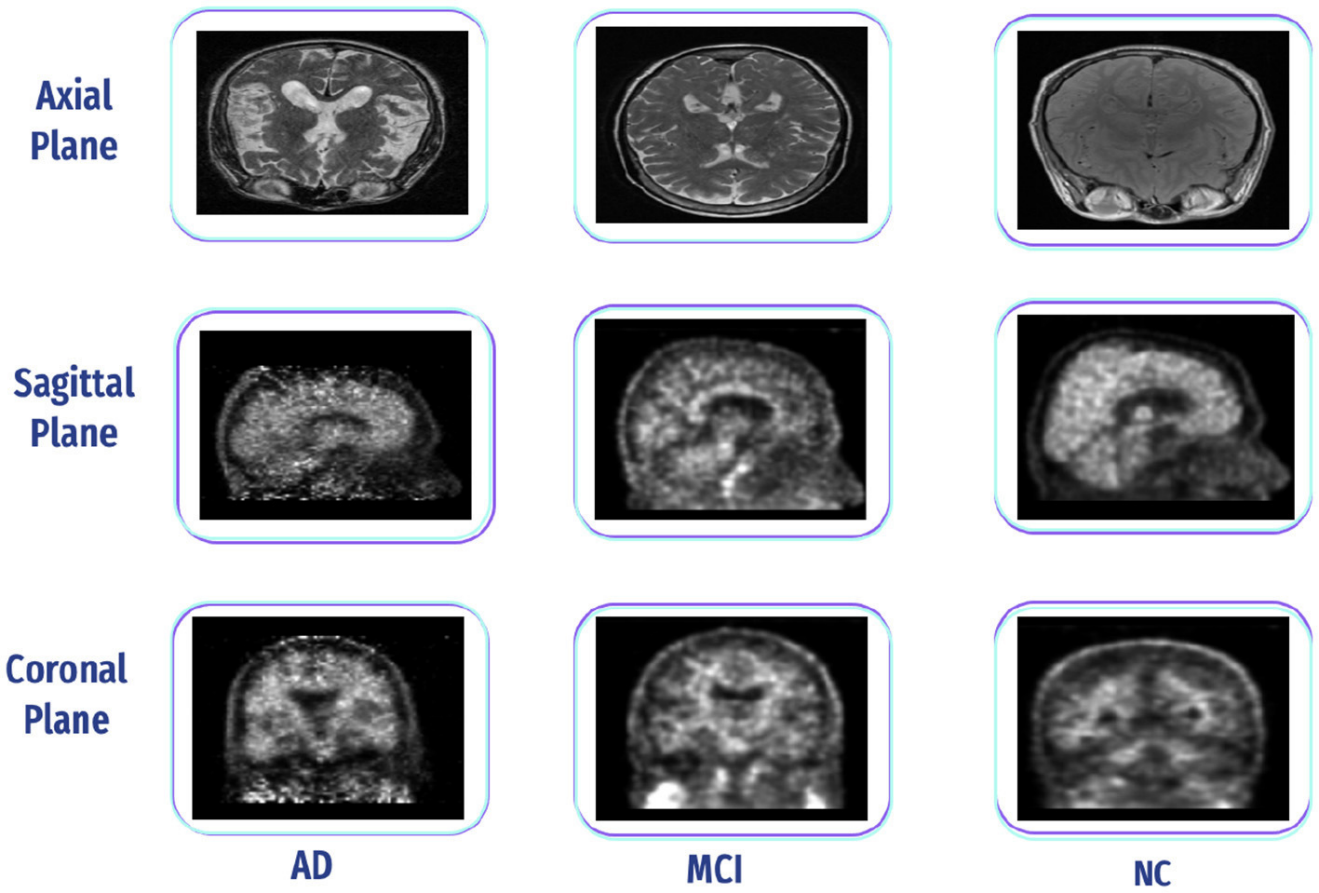
Three-plane view of AD, CN, and MCI from the ADNI dataset.

Its free-access dataset is retrieved from the ADNI. The ADNI was initiated in 2004 under the leadership of Dr. Michael W. Weiner. ADNI is a collaborative effort involving multiple institutions and researchers in the United States and Canada. It is a longitudinal study that was carried out in stages at several centers in North America (ADNI1, ADNIGO, ADNI2, and ADNI3). ADNI aims to develop biomarkers as clinical trial outcome measures. The ADNI includes MRI, PET, fMRI, and DTI and genetic data sessions at various stages for males and females (77). Additionally, we can select a sagittal, coronal, and axial plane, adding them to data collections and downloading them as NIFTI files, as shown in Figure 16. In this study, we downloaded fMRI data comprising 95 normal, 35 MCI, and 55 AD subjects.

**Figure 17:**
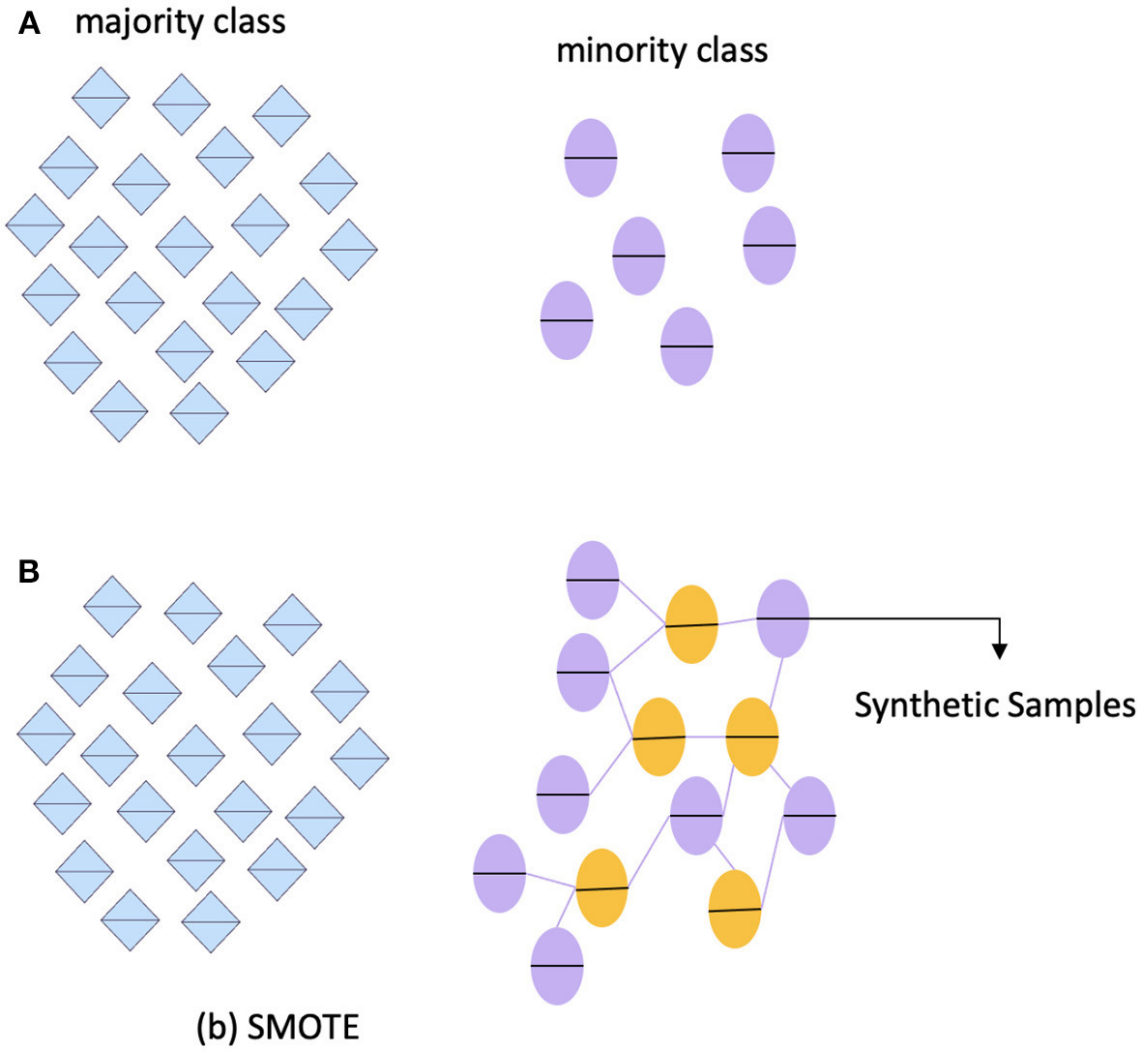
SMOTE techniques to handle imbalanced data. **(A)** imbalance class. **(B)** SMOTE.

Table 3 shows that the ADNI dataset is not imbalanced. However, we still need to address the issue of imbalanced data, as it can affect accuracy. To do this, we utilized the Synthetic Minority Oversampling Technique (SMOTE) (78). One of the most common techniques used to tackle imbalanced data is SMOTE. This technique involves several steps, including identifying the minority classes, selecting their instances, finding the nearest neighbors, and creating synthetic samples. To ensure an equitable representation of participants, the minority class, known as "MCI," was oversampled in this framework. It is important to note that the dataset has no missing or null values, eliminating the need for data imputation or removal. Figure 17 outlines the step-by-step process for predicting AD.

**Table 3:** Subject cohort of fMRI (ADNI dataset).

	**AD**	**MCI**	**NC**
Type of image	NIfTI^*^		
Number of subjects	55	35	95
Total of slices	1096	701	1900
Male/female	30/25	20/15	50/45
Range of age	65–75		
Acquisition plane	Axial rsfMRI (eyes open)		
Voxel size in axial rs-fMRI	3*x*3*x*3*mm*^3^		
TR/TE	TR = 3 s; TE = 30		
Thickness	3.312999963760376 mm		
Acquisition scanner	Philips medical systems		

^*^Neuroimaging informatics technology initiative.

Table 4 shows the main differences between the OASIS and ADNI datasets are as follows: OASIS provides open access to a diverse population but with less comprehensive data, while ADNI provides extensive data and standardized protocols but with restricted access and a more homogeneous population.

**Table 4:** Different between OASIS and ADNI datasets.

	**OASIS**	**ADNI**
Availability	Openly available to the scientific community	available to researchers, but access is subject to data use agreements and restrictions.
Size and Scope	Relatively smaller dataset with fewer subjects and a narrower focus multiple	larger with data from sites and a broader range of assessments.
Cost and resources	Require less computational resources.	Require additional resources expertise due to its comprehensive nature and complex data structure.

### 5.2 Evaluation analysis

The trained model's performance is measured using evaluation metrics, with each implementation having a different preprocessing and classifier training (79).

#### 5.2.1 K-fold cross-validation

**Figure 18:**
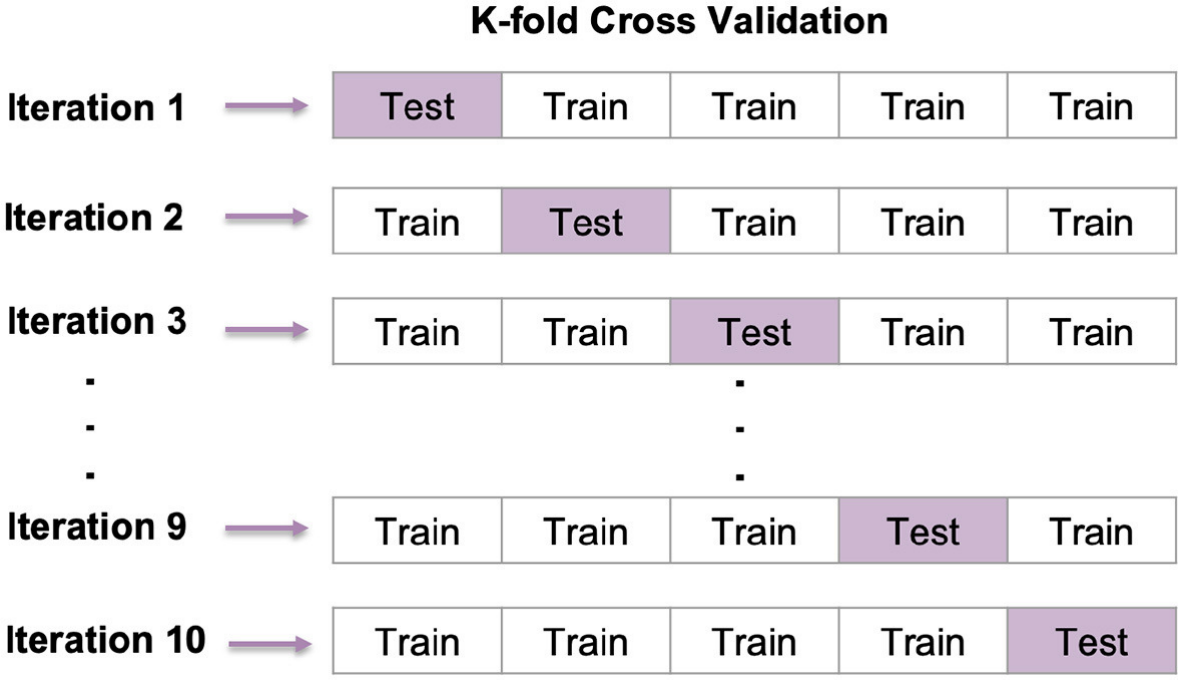
K-fold cross-validation.

Cross-validation is a widely used method in ML for evaluating how well a model can make predictions. This method is easy to understand and helps reduce bias during evaluation. We have used 10-fold cross-validation for each configuration created by combining available values (80), as shown in Figure 18.

#### 5.2.2 Classification metrics

This section presents the experimental results, focusing on precision, recall, F1-score, and accuracy metrics used for disease classification. These metrics are calculated based on confusion metrics, and accordingly, the performance metrics were identified in Equation (5) (81):


(5) $$\begin{array}{l} Precision=TP/\left(TP+FP\right) \end{array}$$

Recall, also referred to as sensitivity (SN), is defined as the capability to identify AD patients. The formula is given in Equation (6):


(6) $$\begin{array}{l} Recall=TP/\left(TP+FN\right) \end{array}$$

F1- score is a degree of the accuracy of the test, which reflects both the recall and precision of the test to calculate the score. The given formula is for the F1- score in Equation (7):


(7) $$\begin{array}{l} F1\text{-}score=2TP/\left(2TP+FP+FN\right) \end{array}$$

Accuracy (ACC) is the likelihood of correct positive and negative forecasts, as shown in Equation (8).


(8) $$\begin{array}{l} ACC=TP+TN/TP+TN+FP+FN \end{array}$$

Where the parameters TP, FP, TN, and FN are defined as follows:

- True positive (TP): The subject has AD, and categorization outcome is positive (AD).
- False positive (FP): A subject has NC, and categorization outcome is positive.
- True negative (TN): A subject has NC, and categorization outcome is negative (Normal).
- False negative (FN): The person has AD, but the test is negative.

## 6 Comparison with previous studies

Based on the related study presented in Table 5, it was observed that most studies depend on a single model for ML and DL to classify AD. Moreover, most of the studies used a small dataset and the AAL-90 atlas to define the nodes (regions) of the brain. Thus, it is necessary to improve a model in various ways to extract and select essential features. Our study used MVPA for fMRI scans from the OASIS-3 and ADNI datasets to extract activation and connectivity patterns.

**Table 5:** Comparison between our study and similar studies in the fMRI literature.

				**Type of class**			
**References**	**Perf**	**Data**	**Model**	**AD/MCI**	**AD/NC**	**MCI/NC**	**AD/MCI/NC**
Lama and Kwon (28)	Acc		LSVM	-	-	-	96.11%
	f1-Sc		-	-	-	-	97.3%
	Rec	ADNI	-		-	-	95.03%
	Prec			-	-	-	97.18%
Parmar et al. (29)	Acc			-	94.58%	-	-
	f1-Sc				94.82%	-	-
	Rec	ADNI	3D-CNN	-	95.2%	-	-
	Prec			-	94.44%	-	-
Guo and Zhang (30)	Acc			-	-	-	-
	f1-sco			-	-	-	-
	Rec	ADNI-2	AE	-	94.6%	-	-
	Pre			-	96.7%	-	-
Shahparian et al. (32)	Acc			98.26%	97.51%	-	-
	f1-sco			98.9%	98.28%	-	-
	Rec	ADNI	SVM	97.83%	100%	-	-
	Pre			100%	96.63%	-	-
Wang and Lim (34)	Acc			72.7%	84.8 %	97.7%	-
	f1-sco			-	-	-	-
	Rec	ADNI	ZNN	-	-	-	-
	Pre			-	-	-	-
Jiao et al. (37)	Acc			-	-	91.13%	-
	f1-sco			-	-	-	-
	Rec	ADNI-2	SVM	-	-	93.17%	-
	Pre			-	-	87.92%	-
Yang et al. (39)	Acc			-	-	91.13%	-
	f1-sco			-	-	-	-
	Rec	ADNI-2	SVM	-	-	93.17%	-
	Pre			-	-	87.92%	-
Begum and Selvaraj (44)	Acc			97.52%	97.53%	-	-
	f1-sco			97.14%	98.46%	-	-
	Rec	ADNI	3D-DCNN	90.48%	95.42%	-	-
	Pre			94.98%	97.98%	-	-
	Acc			83.3%	**93.18%**	92.15%	87.79%
	f1-sco			83.44%	**93.28%**	92.44%	88.64%
	Rec	ADNI	SVM	83.30%	**93.28%**	92.15%	87.79%
	Pre			83.3%	**94.29%**	**93.99%**	**92.60%**
Our work	Acc			84%	88.71%	89.57%	88.28%
	f1-sco			84.21%	88.83%	**90%**	88.41%
	Rec	ADNI	KNN	84%	88.71%	**89.57%**	88.28%
	Pre			88%	**89.96%**	**89.96%**	**90.34%**
	Acc			87.92%	**89.98%**	80%	**91%**
	f1-sco			87.94%	**89.52%**	81.19%	**91%**
	Rec	ADNI	Adaboost	87.92%	89.98%	80%	89%
	Pre			88.53%	**91.39%**	88.38%	**92%**
	Acc			**94.93%**	**90.94%**	**96.15%**	**93.96%**
	f1-sco			**94.98%**	91%	**96.23%**	**94%**
	Rec	ADNI	*HML	**94.93%**	**90.94%**	**96.15%**	**93.96%**
	Pre			**95.61%**	**93%**	**96.65%**	**94.78%**
	f1-sco			86.49%	80.49%	**90.51%**	88%
	Rec	OASIS	SVM	86.97%	81.33%	90%	85%
	Pre			**90.79%**	89 %	**90%**	**91%**
	Acc			86.4%	86%	89%	84%
	f1-sco			86.26%	86.8%	**90.5%**	84%
	Rec	OASIS	KNN	86.47%	86.7%	89%	77%
	Pre			87.58%	**89.8%**	89.8%	**92%**
	Acc			82.94%	86.16%	**89.94%**	87%
	f1-sco			82.9%	86%	82.9%	87%
	Rec	OASIS	Adaboost	82.94%	86.1%	89.94%	86%
	Pre			82.93%	**89.75%**	89.30%	88%
	Acc			**95.47%**	**95.11%**	**93.5%**	**92%**
	f1-sco			**95.50%**	**95.13%**	**93.54%**	92%
	Rec	OASIS	HML	**95.47%**	**95.11%**	**93.49%**	93%
	Pre			**96.22%**	**95.83%**	**93.34%**	**92%**

^*^ Hybrid machine learning. Bold values indicate highest score.

## 7 Conclusion

AD is referred to as a neurodegenerative disease that worsens gradually and irreversibly over time. In this article, we proposed a framework to compute FC through MVPA. The fMRI data are relatively complex, with numerous voxels representing different brain regions in 3D space. We used LASSO to select a subset of relevant voxels for a specific analysis to simplify the analysis and focus on the most critical voxels.

We defined the ROIs or brain areas to analyze FC. These ROIs are often selected based on previous knowledge or hypotheses. Moreover, time-series data were extracted from these ROIs. For MVPA, the activity across multiple voxel patterns is crucial. Each data point represents the activity pattern in a specific ROI for a given task, and a correlation matrix of fMRI data is then computed. We applied our framework to single and HML algorithms to classify AD stages based on the activity patterns within ROIs. Our method surpasses state-of-the-art techniques in identifying AD, MCI, and NC in the experimental results.

Medical image classification is a crucial issue in computer science that has been extensively studied over recent decades. While significant improvement has been made in the reliability of various methods, they may need to provide accurate results due to their limitations in terms of universality, susceptibility to illumination effects, and the inadequacy of data quality, resulting in poor accuracy. We have many dimensions, few data points for each scan, and the training sample in fMRI. Additionally, trades between having enough non-redundant features to capture and not having too many noise features lead to overfitting on our data; so it is hard to distinguish between a noise and a signal accurately. In addition, we applied AAL3 to extract the ROI that includes 170 regions, but in preprocessed and defined regain, it only used 166 regions, and some regain skipped. Finally, the variety of public datasets is not that wide. Additionally, we posed the problem as an fMRI scan in all of our experiments. The main obstacle remains the intricate nature of the data and the restricted sample size within the existing dataset.

In the future, we intend to improve early detection performance by employing advanced AI methods such as explainable AI (XAI), to provide explainable results, in addition to label predictions. Moreover, we will extend the framework to track different disease modalities, such as PET and MRI. Moreover, we aim to increase the number of stages to include all the stages of AD, such as EMCI and LMCI.

## Data availability statement

The original contributions presented in the study are included in the article/supplementary material, further inquiries can be directed to the corresponding author.

## Author contributions

BA: Conceptualization, Formal analysis, Project administration, Validation, Writing - review & editing. MA: Conceptualization, Data curation, Methodology, Software, Visualization, Writing - original draft.
